# English Longitudinal Study of Ageing: Associations Between Common Mental Disorder and Oral Health

**DOI:** 10.1111/odi.70124

**Published:** 2025-11-24

**Authors:** Afshan Mirza, Richard G. Watt, Anja Heilmann

**Affiliations:** ^1^ Department of Epidemiology and Public Health University College London London UK

**Keywords:** common mental disorder, GHQ‐12, mental health, OHRQOL, oral health

## Abstract

**Aim:**

To assess the relationship between common mental disorder and oral health in a representative sample of older adults in England.

**Methods:**

We analyzed cross‐sectional data from 8620 individuals aged 50 years and over who participated in the English Longitudinal Study of Ageing, wave 3 (2006–2007). Common mental disorder (no/yes) was assessed using the 12‐Item General Health Questionnaire (GHQ‐12). Oral health outcomes were oral impact on daily performance (no impacts/at least one impact), self‐rated oral health (excellent; very good; good/fair; poor), and edentulousness (dentate/edentate). Associations were assessed using Poisson regression with robust variance, controlling for demographic, socioeconomic, and health factors.

**Results:**

Reporting at least one oral impact on daily performance was more prevalent among those with a common mental disorder than those without (PR = 1.60; 95% CI 1.32–1.96), as was reporting fair/poor self‐rated oral health (PR = 1.23; 95% CI 1.09–1.40) in fully adjusted analyses. We found no association between common mental disorder and edentulousness.

**Conclusion:**

In this sample of UK older adults, common mental disorder was associated with poorer oral health. To promote healthy ageing, older adults with a common mental disorder should be supported to maintain good oral health.

## 1 Introduction

Mental disorders and oral diseases are prevalent public health problems (WHO 2023, 2025); however, less is known about the link between these conditions (Kenny et al. 2020; Kisely 2016). Oral health is an important component of overall health, allowing fundamental processes of daily life such as chewing, speaking, and smiling to occur without pain, discomfort, and embarrassment. However, for those with mental disorders, oral health remains a neglected component of care (Kenny et al. 2020; Kisely 2016).

The term common mental disorder refers to prevalent, non‐psychotic mental health conditions such as depression and generalized anxiety disorders (National Institute of Clinical Excellence 2011). Typically characterized by low mood, negative thought patterns, and excessive worry, common mental disorder may be associated with challenges to everyday routines and self‐care practices (McManus et al. 2016). Whilst a common mental disorder can occur at any point over the life course, older adults are exposed to complex physiological and psychosocial factors that may increase the risk of mental ill health. Globally, it is estimated that 14% of adults aged 60 years and over are affected by a mental disorder (WHO 2023). Physical and neurological symptoms associated with frailty, multimorbidity, and cognitive impairment may pose a significant risk to mental health in older adults (WHO 2023). Psychosocial factors such as retirement, loss of financial independence, and reduced social participation may also be linked to mental disorders in this population (Dang et al. 2022). Furthermore, due to population ageing, the number of older adults with a common mental disorder is expected to increase (Rong et al. 2024).

Older adults may also face additional complex barriers to good oral health as a consequence of the ageing process. Long‐term illness, multimorbidity, and cognitive decline may impact behaviours such as oral hygiene routines, dietary choices, and access to dental services (Public Health England 2016). Xerostomia due to age or polypharmacy and sugar‐based medications may also contribute to dental disease in older adults (Cannon et al. 2023).

A number of behavioural and physiological pathways between mental and oral health have been suggested. Those with a common mental disorder may experience a lack of motivation and poorer self‐efficacy, which may negatively impact oral health related behaviours (Skośkiewicz‐Malinowska et al. 2018; Okoro et al. 2012). Coping strategies may include smoking (Fluharty et al. 2017) and increased sugar consumption (Skośkiewicz‐Malinowska et al. 2018). Xerostomia, a risk factor for caries, may occur as a direct symptom of a common mental disorder (Gholami et al. 2017) or as a side effect of medication (Cannon et al. 2023). In addition, it is likely that the relationship between common mental disorder and oral health is bi‐directional (Kisely et al. 2016).

A systematic review and meta‐analysis of common psychological disorders and clinical oral health outcomes by Kisely et al. (2016) included two studies on older adult populations (Anttila et al. 2001; Persson et al. 2003). Meta‐analysis found a positive association between common psychological disorder and a higher risk of decayed, missing, and filled tooth surfaces, mean difference 3.20 (95% CI 1.24–5.17) and tooth loss (OR 1.22; 95% CI 1.14–1.30). Another systematic review by Cademartori et al. (2018) reported higher odds of edentulism among those with depression (pooled OR = 1.17; 95% CI 1.02–1.34).

Associations between mental health and subjective oral health outcomes among older adults have been assessed by a smaller number of studies, focusing mainly on depression. Links between depression and poorer oral health‐related quality of life were identified in a longitudinal study from South Korea (Nerobkova et al. 2023) and a small study from Japan (Noguchi et al. 2017). In the UK, depression has been linked to poorer self‐rated oral health (OR = 1.38; 95% CI 1.01–1.89) among those aged 50 years and over (Venturelli et al. 2021). Similarly, in a representative cohort of Norwegian older adults, higher odds of poorer self‐rated oral health were reported for those who experienced psychological distress (OR = 1.89; 95% CI 1.14–3.15) (Dahl et al. 2018). So far, no UK study has examined the link between the broader concept of common mental disorder and oral health among older people.

In line with the Decade of Healthy Ageing (2021–2030), there is an urgent call to action from political, social, and health perspectives to improve the quality of life of older adults (United Nations 2022). Given the importance of good oral health in later life, it is important and timely to further expand research on the links between mental and oral health among this population group and to shed light on this often neglected area of wellbeing (Johnson et al. 2025).

The aim of this cross‐sectional study was to assess the relationship between common mental disorder and three oral health outcomes (oral health related quality of life, self‐rated oral health, and edentulousness) in a representative older adult population in England. It was hypothesized that common mental disorder is associated with worse oral health.

## 2 Material and Methods

The reporting of this study follows STROBE guidance (Table S1).

### 2.1 Data

We analyzed cross‐sectional data from the English Longitudinal Study of Ageing (ELSA 2024) wave three (2006–2007). The ELSA study commenced in 2002 and is a representative cohort study of older adults aged 50+ years and their partners living in England. The original sample was recruited from the Health Survey for England (HSE) in 1998, 1999, and 2001, and participants were subsequently followed up at 2‐year intervals. Wave three of the study included a refreshment sample of adults aged 50–53, selected from participants in HSE survey years 2001 to 2004. The analytical sample was restricted to community‐dwelling core members aged 50 years and over. All data used in this study were self‐reported and collected by computer‐assisted personal interviewing (CAPI). Ethical approval for ELSA was obtained from the National Research and Ethics Committee, and all participants provided informed consent. ELSA is funded by the US National Institute of Ageing and the UK government.

### 2.2 Outcome Variables: Oral Impact on Daily Performance (OIDP), Self‐Rated Oral Health (SROH) and Edentulousness

Two subjective oral health measures are available in ELSA wave three: Oral Impact on Daily Performance (OIDP), a measure of oral health related quality of life, and self‐rated oral health (SROH). ELSA uses a shortened version of the OIDP questionnaire examining the impact of oral health on five daily functional and social areas (Tsakos et al. 2001). The OIDP instrument asks whether: “*In the past 6 months, have any problems with your mouth, teeth or dentures caused you to have any of the following?*” Difficulty eating food; difficulty speaking clearly; problems with smiling, laughing and showing teeth without embarrassment; problems with emotional stability; problems enjoying the company of other people such as family, friends or neighbours. Response options for each item are yes or no. A response category for no impact is also provided. A dichotomous variable was created distinguishing between those reporting no impacts (coded 0) and those reporting at least one impact (coded 1). SROH was assessed with the following question: “*In relation to your dental health, which of the following applies?*” and dichotomized into 0 = excellent/very good/good and 1 = fair/poor. ELSA also collected data on natural teeth present. From these data, the sample was categorized as 0 = dentate and 1 = edentulous.

### 2.3 Exposure Variable

Common mental disorder was assessed using the 12 Item General Health Questionnaire (GHQ‐12), available in ELSA wave three only. Developed by Goldberg and Williams (1988), the GHQ‐12 questionnaire is a shortened version of the original General Health Questionnaire, and is commonly used as a community screening tool to identify the presence of a common mental disorder (Knüppel et al. 2017; Böhnke and Croudace 2016). The GHQ‐12 questionnaire determines current mental state with 12 questions related to anxiety/depression, perceived stress, loss of confidence and general happiness. The GHQ‐12 questionnaire asks participants: “*Have you recently …* been able to concentrate on whatever you're doing; lost much sleep over worry; felt you were playing a useful part in things; felt capable of making decision; felt constantly under strain; felt you couldn't overcome your difficulties; been able to enjoy your normal day to day activities; been able to face up to your problems; been feeling unhappy or depressed; been losing confidence in yourself; been thinking of yourself as a worthless person; and been feeling reasonably happy all things considered”?. Responses for each question were given on a four‐point ordinal scale (1, 2, 3, 4) and re‐coded using a bimodal scoring system (0, 0, 1, 1) (Noguchi et al. 2017). Response categories were further dichotomized to 0 and 1. The 12 individual questions were summed together to obtain an overall score ranging from 0 to 12. A binary variable was derived with a score of four or more indicating presence of a common mental disorder (Morris and Earl 2017).

### 2.4 Covariates

Similar to previous studies (Dahl et al. 2018; Delgado‐Angulo et al. 2015), the following confounders were included in the analyses: sex (male/female), age (50–64; 65–74; and 75+ years), ethnicity (white/non‐white), marital status (married/partner; single), long‐standing illness (yes/no), self‐reported health (very good; good; fair; bad; and very bad), and smoking status (smoker; non‐smoker; and ex‐smoker). Education (higher degree/equivalent; higher education/A‐Level/no degree; secondary education/other; and no qualifications) and non‐housing wealth quintiles were selected as socioeconomic markers.

### 2.5 Statistical Analysis

Missing data were inspected for differences between those with and without missing observations. Due to a substantial amount of missingness in the GHQ‐12 measure (17.6%), we used multiple imputation by chained equations to minimize potential selection bias. The imputation model included all study measures, as well as the CASP‐19 scale and a binary indicator for doctor‐diagnosed depression as auxiliary variables. Twenty‐five imputed datasets were created. Only participants with observed outcome data were analyzed, resulting in a final analytical sample of 8620 participants. Sample characteristics are shown for observed and imputed data (Table 1), and all other results are reported for imputed data.

**TABLE 1:** Weighted sample characteristics in the analytical sample, observed, and imputed data (ELSA Wave 3, 2006–2007), *N* = 8620.

	Observed		Imputed
	*n*	%	%
OIDP			
No impact	7882	91.3	91.3
At least one impact	738	8.7	8.7
SROH			
Excellent/very good/good	7047	81.2	81.2
Fair/poor	1573	18.8	18.8
Edentulousness			
No	7174	82.4	82.4
Yes	1446	17.6	17.6
Common mental disorder			
No	6233	86.2	85.4
Yes	985	13.8	14.6
Missing	1402		
Sex			
Male	3856	46.8	46.8
Female	4764	53.2	53.2
Age			
50–64	4426	52.4	52.4
64–75	2270	25.4	25.4
75+	1924	22.2	22.2
Ethnicity			
White	8390	96.7	96.7
Non‐White	227	3.3	3.3
Missing	3		
Marital status			
Married/partner	5618	65.9	65.9
Single	3002	34.1	34.1
Education			
Degree/equivalent	1467	15.3	15.4
Higher education/a level	1960	21.5	21.6
Secondary education/other	2637	30.5	30.6
No qualifications	2552	32.4	32.4
Missing	4		
Non‐housing wealth (quintiles)			
Least wealthy	1553	19.9	19.9
2	1613	19.8	19.8
3	1697	20.2	20.2
4	1723	19.9	20.0
Most wealthy	1747	20.1	20.2
Missing	287		
Long standing illness			
No	3855	45.3	45.3
Yes	4759	54.6	54.7
Missing	6		
Self‐rated general health			
Very good	2149	24.4	24.4
Good	3684	42.4	42.4
Fair	2176	25.6	25.8
Bad	487	5.9	5.9
Very bad	121	1.5	1.5
Missing	3		
Smoking status			
Current smoker	1303	15.8	15.8
Ex‐smoker	542	6.1	6.1
Non‐smoker	6774	78.0	78.1
Missing	1		

We first examined cross‐tabulations between each oral health outcome, the exposure (common mental disorder), and covariates (Table 2). We then estimated a series of robust Poisson regression models to assess associations between GHQ‐12 and each oral health outcome (Table 3), controlling for age/sex (Model 1), marital status/ethnicity (Model 2), socioeconomic factors (Model 3), general health (Model 4), and smoking (Model 5) in line with the hierarchical approach to modelling proposed by Victora et al. (1997). We report prevalence ratios (PR) and 95% confidence intervals.

**TABLE 2:** Oral health outcomes by exposure and covariates, weighted estimates using imputed data (*N* = 8620).

	OIDP (at least one impact) (%)	SROH fair/poor (%)	Edentulousness (%)
Common mental disorder			
No	7.3	16.9	17.1
Yes	17.1	30.3	20.5
Sex			
Male	8.6	20.4	14.9
Female	8.8	17.4	19.9
Age			
50–64	7.9	20.4	6.8
64–75	9.8	17.3	19.8
75+	9.6	16.9	40.5
Ethnicity			
White	8.6	18.6	17.7
Non‐White	1.4	26.3	15.1
Marital status			
Married/partner	7.8	16.9	13.2
Single	10.6	22.7	26.1
Education			
Degree/equivalent	6.9	15.4	4.3
Higher education/a level	8.0	17.4	9.8
Secondary/other	8.0	17.6	14.6
No qualifications	11.0	22.6	31.9
Non‐housing wealth (quintiles)			
Least wealthy	12.5	27.8	25.7
2	10.0	20.7	27.0
3	8.2	17.1	17.1
4	6.5	14.6	11.9
Most wealthy	6.5	14.1	6.5
Long standing illness			
No	5.0	14.8	12.9
Yes	11.8	22.1	21.5
Self‐rated general health			
Very good	3.9	0.90	10.1
Good	6.9	15.3	14.6
Fair	13.4	28.0	25.6
Bad	16.9	36.3	28.8
Very bad	27.0	51.0	42.8
Smoking status			
Smoker	13.7	28.5	24.8
Ex‐smoker	9.5	24.2	10.7
Non‐smoker	7.7	16.4	16.7

**TABLE 3:** Prevalence ratio (PR) estimates for Poisson regression models assessing the association between common mental disorder and oral health outcomes, weighted analysis using imputed data (*N* = 8620).

	PR (95% CI)				
	Model 1	Model 2	Model 3	Model 4	Model 5
OIDP	2.35 (1.97–2.81)***	2.25 (1.88–2.70)***	2.13 (1.76–2.57)***	1.62 (1.33–1.97)***	1.60 (1.32–1.96)***
*E*‐value					2.58
SROH	1.82 (1.62–2.05)***	1.74 (1.54–1.95)***	1.62 (1.43–1.82)***	1.24 (1.09–1.41)***	1.23 (1.09–1.40)***
*E*‐value					1.77
Edentulousness	1.17 (1.03–1.36)*	1.15 (1.00–1.32)*	1.04 (0.91–1.20)*	0.91 (0.79–1.05)	0.90 (0.79–1.05)

*Note:* Model 1: adjusted for age and sex; Model 2: Model 1 + ethnicity and marital status; Model 3: Model 2 + education and income; Model 4: Model 3 + self‐rated general health and long standing illness; Model 5: Model 4 + smoking.

* *p* < 0.05.

*** *p* < 0.001.

Poisson regression with robust standard errors is one of the preferred methods for common binary outcomes in cross‐sectional or cohort studies since odds ratios from logistic regression can overestimate the true prevalence ratio or relative risk (Chen et al. 2018; Barros and Hirakata 2003). Statistical analyses were conducted in STATA version 18.5 (StataCorp 2023). All descriptive and regression analyses were run using STATA's suite of ‐svy‐ commands and appropriate ELSA survey weights to account for the complex survey design and unequal probability of being sampled.

Sex, age, and smoking were also considered as potential effect modifiers (Piccinelli and Wilkinson 2000; Fluharty et al. 2017). Effect modification was tested by separately including interaction terms in the regression models. Preliminary analyses found no evidence for interactions (adjusted Wald test *p*‐value > 0.05).

A sensitivity analysis was carried out with Poisson regression models run on complete data (*N* = 6979) for comparison (Table S2). Using estimates from the fully adjusted models, *E*‐values were calculated as a measure of residual confounding: $\mathrm{RR}+\sqrt{\mathrm{RR}}x\left(\mathrm{RR}-1\right)$. *E*‐values provide an estimate of how large an unmeasured confounder would need to be to fully explain the associations, conditional on the measured covariates (VanderWeele and Ding 2017).

## 3 Results

The final analytical sample included 8620 participants (Figure S1). Mean age was 65.0 years (SD ± 0.14). Table 1 provides sample characteristics for observed and imputed data. In the following we report results for imputed data. The prevalence of a common mental disorder was 14.6%. 18.8% of the sample rated their oral health as fair/poor, 17.6% were edentulous and 8.7% reported at least one oral impact. The most common oral impact was difficulty eating food (11.7%) followed by problems with smiling, laughing and showing teeth without embarrassment (5.5%) (Table S3).

The distribution of oral health outcomes by exposure and covariates are shown in Table 2. Compared to those without, participants with a common mental disorder were more likely to report at least one oral impact (17.1% vs. 7.3%); rate their oral health as fair/poor (30.3% vs. 16.9%) and be edentulous (20.5% vs. 17.1%). Associations with education and wealth followed a clear social gradient—those with lower levels of education and wealth were more likely to report poorer oral health outcomes.

Table 3 presents the results of Poisson regression models assessing associations between common mental disorder and oral health outcomes. After adjusting for demographic and socio‐economic factors, moderate attenuation was observed in effect size for reporting at least one oral impact. After controlling for health variables, further marked attenuation was observed. In the final adjusted model, having a common mental disorder was associated with a 60% higher prevalence of reporting at least one oral impact (PR = 1.60; 95% CI 1.32–1.96). Results for self‐rated oral health followed a similar pattern to oral impact on daily performance. After full adjustment, those with a common mental disorder had 1.23 (95% CI 1.09–1.40) times the prevalence of reporting fair/poor self‐rated oral health. Finally, those with a common mental disorder were 1.17 times more likely to be edentulous compared to those without. However, after adjustment for all covariates the association was fully explained (PR = 0.90; 95% CI 0.79–1.05).

After controlling for all covariates, *E*‐values of 2.58 were calculated for oral impact on daily performance and 1.77 for self‐reported oral health. Based on the reported *E*‐values, estimates for oral impact on daily performance appear robust.

## 4 Discussion

The findings of this study support our hypothesis that older adults with a common mental disorder are at higher risk of poor oral health. The prevalence of reporting at least one oral impact or poorer self‐rated oral health was higher (60% and 23%, respectively) among those with a common mental disorder than those without, after controlling for covariates. Having a common mental disorder was not significantly associated with edentulousness.

Effect sizes for oral impact on daily performance and self‐rated oral health were moderate, which is broadly in line with previous studies on depression and subjective oral health (Dahl et al. 2018; Venturelli et al. 2021). However, given the high prevalence of poor mental health and projected rise of mental health issues among older adults in an ageing population (Rong et al. 2024), from a population health perspective, these small to moderate effect sizes may contribute to a significant overall burden.

ELSA does not collect clinical data; however, subjective oral health measures are strongly related to clinical dental health (Blizniuk et al. 2017). Subjective health measures provide professionals and policymakers with an understanding of disease impact from a quality of life perspective (Ueno et al. 2015). Self‐rated oral health is strongly associated with oral conditions such as dental caries, periodontal disease, and tooth loss (Blizniuk et al. 2017; Ueno et al. 2015). Consequently, study findings would suggest that older adults with a common mental disorder are at higher risk of untreated dental disease, as well as pain and discomfort associated with poor oral health. In this study, nearly one in five older adults (17.1%) with a common mental disorder reported at least one impact on daily performance. Those with a common mental disorder were more likely to report difficulties with basic activities such as eating and smiling without embarrassment. Consequently, the impact of poor oral health on functional and social aspects of quality of life must be considered from a healthy ageing perspective.

Whilst this study has interpreted common mental disorder as a risk factor for worse oral health, a bi‐directional relationship is plausible and supported by previous evidence (Kisely 2016). In addition to the potential pathways from mental to oral health set out in the introduction, oral health issues linked to pain, reduced quality of life, and social embarrassment may also influence mental health outcomes (Campos et al. 2020; Skośkiewicz‐Malinowska et al. 2018).

For both oral impact on daily performance and self‐rated oral health, socioeconomic markers explained part of the association between common mental disorder and worse oral health. This is not surprising, considering the body of evidence linking social deprivation to both oral and mental health (Venturelli et al. 2021; WHO 2023). Controlling for general health indicators resulted in further substantial attenuation of effect sizes, suggesting additional shared pathways. Our findings highlight the importance of a Common Risk Factor Approach that aims to address the underlying structural and intermediate determinants of health overall (Watt and Sheiham 2012; Solar and Irwin 2010).

We found no association between common mental disorder and edentulousness. In ELSA, it is not possible to determine exactly when or why tooth loss occurred. Tooth loss is the result of chronic, cumulative disease processes and is often the end point of dental caries and periodontal disease (Murray Thomson 2014). Consequently, further longitudinal studies are needed to examine associations between mental health and edentulousness over the life course to gain a better understanding of this relationship.

The results of this study support previous research reporting associations between common mental disorder and worse subjective oral health measures in older adult populations (Dahl et al. 2018; Noguchi et al. 2017). Similar to our study, Noguchi et al. (2017) found that among a small sample of older people in Japan (*N* = 187), those with a higher GHQ‐12 score were more likely to report worse oral health related quality of life. The findings are also in line with previous reports of no association between common mental disorder and edentulousness (Hybels et al. 2016; Roohafza et al. 2015; Wiener et al. 2015). We identified only one comparable UK study (Venturelli et al. 2021), also based on ELSA data, which similar to our study reported an association between mental health (depression) and poor self‐rated oral health (OR = 1.38; 95% CI 1.01–1.89), but not with edentulousness. In contrast to our findings, depression was not linked to oral impacts on daily performance, possibly due to a considerably smaller sample size (*N* = 3617) limiting statistical power.

Our results differ from the conclusion of a systematic review that reported depression as a risk factor for edentulousness (Cademartori et al. 2018). However, studies included in the systematic review were based on significantly larger non‐UK populations (Okoro et al. 2012; Saman et al. 2014).

A major strength of this study was the use of a large, representative sample. We adjusted for a range of potential confounders including two measures of socioeconomic status, education and wealth. However, it is possible that study findings are impacted by residual confounding as factors such as diet, oral hygiene, and dental attendance could not be considered because they are not available in ELSA. We have calculated *E*‐values to provide an estimate of how large an unmeasured confounder would need to be to fully explain the associations. Other study limitations include the low proportion of non‐white participants, questioning the generalizability of results to this group. Furthermore, as data on common mental disorder and oral health were self‐reported, the issue of reporting bias must also be considered. Having a common mental disorder might influence perceptions of other health aspects, potentially resulting in an overestimation of effect sizes.

Our study adds to the existing evidence on the links between mental and oral health. There is an urgent need to address oral health within mental health practice. Current policies on mental health fail to recognize the importance of oral health and lack clear and consistent oral health guidance (Johnson et al. 2025). Consequently, the integration of oral health within a broader mental health framework provides an opportunity for health and social care practitioners to deliver cohesive and collaborative guidance on oral health promotion and disease prevention (Chan et al. 2023). Appropriate training should be provided to mental health professionals on oral health (Johnson et al. 2025). A simple oral health screening tool within mental health assessments and personal care plans can provide useful information on oral hygiene practices, dental attendance, and oral health‐related quality of life, and can help to identify areas of need for prevention and treatment.

Whilst this study presents new evidence on the relationship between mental and oral health among UK older adults, further research is urgently needed. Data on clinical outcomes; oral health related behaviours; and preventative interventional studies on improving oral health can further our understanding of the interrelated nature of mental and oral health in the older adult population. Furthermore, for older adults with a common mental disorder living in care homes and residential facilities, this area of research is particularly important.

In conclusion, whilst the limitations of this study are acknowledged, our findings add to existing evidence for the links between mental and oral health. Healthcare professionals have a duty of care to their patients and this must also extend to their oral health. It is important and necessary that oral health is given attention within mental health policies to support the active ageing agenda.

## Author Contributions

**Afshan Mirza:** conceptualization, investigation, writing – original draft, methodology, writing – review and editing, formal analysis, data curation, software. **Richard G. Watt:** conceptualization, supervision. **Anja Heilmann:** conceptualization, methodology, writing – review and editing, software, data curation, supervision.

## Conflicts of Interest

The authors declare no conflicts of interest.

## Supporting information


**Table S1:** odi70124‐sup‐0001‐Supinfo.docx.

## Data Availability

The data that support the findings of this study are available from UK DATA SERVICE. Restrictions apply to the availability of these data, which were used under licence for this study. Data are available from https://ukdataservice.ac.uk with the permission of UK DATA SERVICE.
