# Supplementary material for: English Longitudinal Study of Ageing: Associations Between Common Mental Disorder and Oral Health
Source: Oral Dis. 2025 Nov 24;32(4):1161–9. doi: 10.1111/odi.70124 (PMC13248586; doi:10.1111/odi.70124)
Supplement: Supplementary file 1 — Table S1: odi70124‐sup‐0001‐Supinfo.docx. [file ODI-32-1161-s001.docx]

**Supporting information**

English Longitudinal Study of Ageing: Associations Between Common Mental Disorder and Oral Health

Afshan Mirza^1^, Richard G Watt^1^ and Anja Heilmann^1^**.**

^1^Department of Epidemiology and Public Health, University College London, London, United Kingdom.

**Supporting information Table 1: STROBE Statement.**

|  | Item No | Recommendation |
| --- | --- | --- |
| **Title and abstract** | 1 | (*a*) Indicate the study’s design with a commonly used term in the title or the abstract  Study design included in abstract |
|  |  | (*b*) Provide in the abstract an informative and balanced summary of what was done and what was found  Abstract included |
| Introduction | | |
| Background/rationale | 2 | Explain the scientific background and rationale for the investigation being reported  Introduction section |
| Objectives | 3 | State specific objectives, including any prespecified hypotheses  Introduction section |
| Methods | | |
| Study design | 4 | Present key elements of study design early in the paper  Materials and methods section |
| Setting | 5 | Describe the setting, locations, and relevant dates, including periods of recruitment, exposure, follow-up, and data collection  Materials and methods: Data and analytical sample section  Participant flow diagram: Supporting information Figure 1 |
| Participants | 6 | (*a*) Give the eligibility criteria, and the sources and methods of selection of participants  Materials and methods: Data and analytical sample section  Participant flow diagram: Supporting information Figure 1 |
| Variables | 7 | Clearly define all outcomes, exposures, predictors, potential confounders, and effect modifiers. Give diagnostic criteria, if applicable  Materials and methods: Outcomes; Exposure; Covariates section |
| Data sources/ measurement | 8* | For each variable of interest, give sources of data and details of methods of assessment (measurement). Describe comparability of assessment methods if there is more than one group  Material and methods :Outcome; Exposure; Covariates section  Participant flow diagram: Supporting information Figure 1 |
| Bias | 9 | Describe any efforts to address potential sources of bias  Materials and methods section: Statistical analyses section: multiple imputation used to manage missing data |
| Study size | 10 | Explain how the study size was arrived at  Participant flow diagram: Supporting information Figure 1 |
| Quantitative variables |  | Explain how quantitative variables were handled in the analyses. If applicable, describe which groupings were chosen and why  Materials and methods section: Outcome; Exposure; Covariates section |
| Statistical methods | 12 | (*a*) Describe all statistical methods, including those used to control for confounding  Materials and methods: Statistical analyses section |
|  |  | (*b*) Describe any methods used to examine subgroups and interactions  Materials and methods: Statistical analyses section. Interactions assessed |
|  |  | (*c*) Explain how missing data were addressed  Materials and methods: Statistical analyses section |
|  |  | (*d*) If applicable, describe analytical methods taking account of sampling strategy  Materials and methods: Statistical analyses section |
|  |  | (*e*) Describe any sensitivity analyses  Materials and methods: Statistical analyses section  Analyses on both imputed (Results Table 3) and complete case data supporting information Table 2)  E-Values reported |
| Results | | |
| Participants | 13* | (a) Report numbers of individuals at each stage of study—eg numbers potentially eligible, examined for eligibility, confirmed eligible, included in the study, completing follow-up, and analysed  Participant flow diagram: Supporting information Figure 1  Results section |
|  |  | (b) Give reasons for non-participation at each stage  Materials and methods: Data and analytical sample section  Participant flow diagram: Supporting information Figure 1 |
|  |  | (c) Consider use of a flow diagram  Participant flow diagram: Supporting information Figure 1 |
| Descriptive data | 14* | (a) Give characteristics of study participants (eg demographic, clinical, social) and information on exposures and potential confounders  Results section |
|  |  | (b) Indicate number of participants with missing data for each variable of interest  Results section |
| Outcome data | 15* | Report numbers of outcome events or summary measures  Results section |
| Main results | 16 | (*a*) Give unadjusted estimates and, if applicable, confounder-adjusted estimates and their precision (eg, 95% confidence interval). Make clear which confounders were adjusted for and why they were included  Results section: Table 3 |
|  |  | (*b*) Report category boundaries when continuous variables were categorized  Results section |
|  |  | (*c*) If relevant, consider translating estimates of relative risk into absolute risk for a meaningful time period  n/a |
| Other analyses | 17 | Report other analyses done—eg analyses of subgroups and interactions, and sensitivity analyses  Materials and methods: Statistical analyses section: Analysis for interactions.  Supporting information Table 2: Results provided for complete case analysis  Results section: E – values reported |
| Discussion | | |
| Key results | 18 | Summarise key results with reference to study objectives  Discussion section points 18-21 |
| Limitations | 19 | Discuss limitations of the study, taking into account sources of potential bias or imprecision. Discuss both direction and magnitude of any potential bias |
| Interpretation | 20 | Give a cautious overall interpretation of results considering objectives, limitations, multiplicity of analyses, results from similar studies, and other relevant evidence |
| Generalisability | 21 | Discuss the generalisability (external validity) of the study results |
| Other information | | |
| Funding | 22 | Give the source of funding and the role of the funders for the present study and, if applicable, for the original study on which the present article is based  Statement made |

*Give information separately for exposed and unexposed groups.

**Note:** An Explanation and Elaboration article discusses each checklist item and gives methodological background and published examples of transparent reporting. The STROBE checklist is best used in conjunction with this article (freely available on the Web sites of PLoS Medicine at http://www.plosmedicine.org/, Annals of Internal Medicine at http://www.annals.org/, and Epidemiology at http://www.epidem.com/). Information on the STROBE Initiative is available at www.strobe-statement

**Supporting information Table 2:** Results from Poisson regression models (Prevalence Ratio) to assess the association between common mental disorder and oral health outcomes, complete case analysis (n=6,979).

|  | **PR (95% CI)** | | | | |
| --- | --- | --- | --- | --- | --- |
|  | **Model 1** | **Model 2** | **Model 3** | **Model 4** | **Model 5** |
| OIDP | 2.37  (1.96-2.86)*** | 2.30  (1.90-2.79)*** | 2.16  (1.77-2.65)*** | 1.68  (1.36 -2.08)*** | 1.67  (1.34 -2.07)*** |
|  |  |  |  |  |  |
| SROH | 1.91  (1.68-2.16)*** | 1.84  (1.63-2.09)*** | 1.72  (1.50-1.95)*** | 1.31  (1.14-1.49)*** | 1.30  (1.14-1.49)*** |
|  |  |  |  |  |  |
| Edentulousness | 1.14*  (1.00-1.32) | 1.12  (0.97-1.30) | 1.10  (0.87-1.17) | 0.87  (0.74-1.01) | 0.87  (0.74-1.01) |

*p<0.05, ***p<0.001

Model 1: adjusted for age and sex;

Model 2: Model 1 + ethnicity and marital status;

Model 3: Model 2 + education and income;

Model 4: Model 3 + self-rated general health and long standing illness;

Model 5: Model 4 + smoking.

**Supporting information Table 3:** Percentage of oral impact on daily performance (OIDP) for those with and without a common mental disorder (N=8,620).

| **OIDP** | **Common mental disorder absent**  **(%)** | **Common mental disorder present**  **(%)** |
| --- | --- | --- |
| Difficulty eating food | 5.4 | 11.7 |
| Difficulty speaking clearly | 1.0 | 3.7 |
| Smiling, laughing and showing teeth without embarrassment | 2.2 | 5.5 |
| Problems with emotional stability | 1.0 | 1.7 |
| Problems enjoying the company of others | 0.0 | 1.3 |
